# Proteomic as an Exploratory Approach to Develop Vaccines Against Tick-Borne Diseases Using Lyme Borreliosis as a Test Case

**DOI:** 10.3390/vaccines8030463

**Published:** 2020-08-21

**Authors:** Emilie Talagrand-Reboul, Benoit Westermann, Matthieu A. Raess, Gilles Schnell, Paola Cantero, Cathy Barthel, Laurence Ehret-Sabatier, Benoit Jaulhac, Nathalie Boulanger

**Affiliations:** 1 FMTS, UR7290: Groupe Borrelia, Université de Strasbourg, 67000 Strasbourg, France; talagrandreboul@unistra.fr (E.T.-R.); matthieu.raess@gmail.com (M.A.R.); cbarthel@unistra.fr (C.B.); jaulhac@unistra.fr (B.J.); 2 Laboratoire de Spectrométrie de Masse BioOrganique, Université de Strasbourg, CNRS, IPHC UMR 7178, 67000 Strasbourg, France; westermann.benoit@hotmail.fr (B.W.); gilles.schnell@gmail.com (G.S.); liz-paola.cantero-mendieta@etu.unistra.fr (P.C.); laurence.sabatier@unistra.fr (L.E.-S.); 3 French National Reference Center on Lyme Borreliosis, CHRU, 67000 Strasbourg, France

**Keywords:** tick-borne diseases, Lyme, *Borrelia*, proteomics, skin, markers of infection

## Abstract

Tick-borne diseases affecting humans and animals are on the rise worldwide. Vaccines constitute an effective control measure, but very few are available. We selected Lyme borreliosis, a bacterial infection transmitted by the hard tick *Ixodes*, to validate a new concept to identify vaccine candidates. This disease is the most common tick-borne disease in the Northern Hemisphere. Although attempts to develop a vaccine exist, none have been successfully marketed. In tick-borne diseases, the skin constitutes a very specific environment encountered by the pathogen during its co-inoculation with tick saliva. In a mouse model, we developed a proteomic approach to identify vaccine candidates in skin biopsies. We identified 30 bacterial proteins after syringe inoculation or tick inoculation of bacteria. Discovery proteomics using mass spectrometry might be used in various tick-borne diseases to identify pathogen proteins with early skin expression. It should help to better develop sub-unit vaccines based on a cocktail of several antigens, associated with effective adjuvant and delivery systems of antigens. In all vector-borne diseases, the skin deserves further investigation to better define its role in the elaboration of protective immunity against pathogens.

## 1. Introduction 

Vector-borne diseases (VBDs) represent a major burden for human and animal health. Ticks are hematophagous ectoparasites that transmit a large panel of pathogens such as bacteria, viruses, and parasites. The bacterial infection called Lyme borreliosis is the most prevalent vector-borne infectious disease in the Northern Hemisphere [1]. The disease is a zoonosis that affects wild and domestic animals; humans constitute accidental hosts. Currently, no vaccine is available to prevent the disease in humans, although various vaccines have been developed in the past, mainly based on *Borrelia* lipoproteins, outer surface protein C (OspC) and OspA [2]. OspC, first considered as an ideal vaccine candidate, was selected. OspC is synthetized within the tick during the migration of the spirochete from the gut to the salivary glands [3]. It is also an essential lipoprotein for transmission and dissemination of *Borrelia* in the vertebrate host [4]. However, due to its diversity, protection was mainly strain-specific. Improvement in the vaccine design, using epitopes from different OspC stains, did not lead to development of a vaccine in humans thus far [5]. OspA on the surface of the spirochete is essential for it to bind to the tick midgut using the specific receptor TROSPA (Tick receptor for outer surface protein A). Upon tick blood meal, spirochetes detach from the midgut receptor and switch their surface lipoproteins from OspA to OspC [6]. The idea emerged that by immunizing mice with OspA, specific antibodies against OspA could neutralize the bacteria within the tick, thus blocking transmission to the vertebrate host [7]. Further, the use of this vaccine in human clinical trials proved that the concept of a “transmission-blocking vaccine” could be used to neutralize a pathogen within the tick vector. Although effective, this vaccine tested in the general population was associated with adverse effects in a few patients and the vaccine was withdrawn from the market in 2002 [8]. Recently, a new formulation of OspA vaccine without the epitopes associated with potential autoimmunity problem and containing six different serotypes has been developed. It is presently tested in a phase 2 human clinical trial [9]. Other *Borrelia burgdorferi* proteins were also tested as vaccine candidates, such as decorin-binding protein A (DbpA) and fibronectin-binding protein (BBK32) [10,11].

Other vaccine concepts have been developed to reduce the incidence of Lyme disease in animal reservoir. As this disease is a zoonosis with the bacterium circulating in numerous hosts, vaccines have also been tested in the main animal reservoirs. The wild white-footed mouse, *Peromyscus leucopus*, has been immunized with various formulations of recombinant OspA to reduce nymphal infection prevalence. Various routes of inoculation have been tested, either subcutaneously on small populations of rodents [12] or on a larger scale with baited oral vaccination strategy [13]. Targeting the tick vector by immunization of the vertebrate host with tick saliva proteins is also explored. As the blood meal of hard ticks lasts for several days, tick saliva is essential to this long feeding process. Tick proteins target the pharmacology (coagulation, itching, and pain) and the immunology (complement pathway, cells of innate and adaptive immunity) of the vertebrate host to allow for blood meal completion. Several candidates have been *Rhipicephalus* identified; among them are Subolesin [14], Salp15 (Salivary protein of 15 kDa) [15], ISAC (*Ixodes scapularis* anticomplement) [16], Ixolaris [17], sialostatins [18], and 64TRP (64Truncated *Rhipicephalus* Protein) [19]. Although various strategies have been evaluated, no effective vaccine is available for tick-borne diseases (TBDs), except against tick-borne encephalitis virus [20].

In VBDs, the skin is an essential organ where the arthropod co-inoculates the pathogens and its saliva [21]. Therefore, a vaccine that would induce an immune response in the vertebrate host to neutralize the pathogen in the skin should be effective. We thus developed a new strategy to identify vaccine candidates present in the skin of the vertebrate host. We evaluated proteomics on skin biopsies of *Borrelia*-infected mice to identify such vaccine candidates [22]. In Lyme borreliosis, bacteria are co-inoculated with tick saliva in the dermis where an intense *Borrelia* multiplication occurs, followed by dissemination to the target organs: the heart, the nervous system, and the joints [23]. However, some bacteria persist in the skin, pointing out the major role of the skin as an immune-tolerant organ. In this study, we used a well-established C3H/HeN mouse model of Lyme borreliosis to select *Borrelia* proteins by non-targeted mass spectrometry that could be further tested as candidates in a Lyme vaccine.

## 2. Material and Methods

### 2.1. Mouse Infection and Bacterial Strains

We selected three *Borrelia burgdorferi* sensu stricto (ss) strains: 297 (AC Steere, Boston, MA, USA, isolated from cerebrospinal fluid samples), IBS 19 (B Jaulhac, Strasbourg, France, isolated from an erythema migrans), and BL 642 (CS Pavia, New-York, NY, USA, isolated from blood samples). All strains were cultured in BSK-H complete medium (Sigma-Aldrich, Saint Quentin Fallavier, France) at 33 °C and used at low passage (<7) for mouse infection. Spirochetes were counted and viability was checked using dark-field microscopy.

Three- to four-week old C3H/HeN mice were inoculated with 10^3^ spirochetes in 0.1 mL BSK by intradermal injection in the dorsal thoracic area. The infection kinetics was assessed, and skin samples of mice were collected at various time points after *Borrelia* inoculation: day 3, 5, 7, and 15. An area of approximately 1 cm of mouse skin was collected at the inoculation site and stored at −80 °C.

For infection via tick bites, *Ixodes ricinus* nymphs infected with *Borrelia afzelii* strain NE4049 were generated according to a previously described protocol [14]. Briefly, each mouse was infected with 10 infected nymphs until blood meal completion. The infection rate of nymphs infected with *Borrelia* was 60–80%. For infection with field-collected ticks, three *I. ricinus* females from an endemic area for Lyme borreliosis [24] were fed per mouse. On day 3, the females were removed from the mice, and after 7 days, samples of potentially infected skin were collected. *Borrelia* detection and quantification were performed by PCR.

### 2.2. Quantification of B. burgdorferi DNA by PCR in Mouse Skin

DNA was extracted from skin samples of each individual mouse on a MagNA Pure system (Roche Diagnostics, Meylan, France), and the load of spirochetes in the skin was subsequently estimated by quantitative PCR targeting the flagellin gene (*flaB*) as previously described [23]. Briefly, quantification of *Borrelia*-specific *flaB* gene was performed on a LightCycler system (Roche Diagnostics, Meylan, France). Quantification of the mouse-specific glyceraldehyde-3-phosphate dehydrogenase (*gapdh*) gene was performed on an ABI Prism 7500 instrument (ThermoFisher, Illkirch-Graffenstaden, France), using a commercial kit (TaqMan rodent GAPDH control reagent; ThermoFisher, Illkirch-Graffenstaden,, France). The number of *B. burgdorferi* spirochetes in the mouse skin was standardized to 10,000 *gapdh* gene copies. 

### 2.3. Proteomics

Samples (5 mg) of mouse skin biopsies were manually extracted by Laemmli buffer (200 µL), and proteins (50 µg) were pre-fractionated onto 12% SDS-PAGE as previously described [22]. Gel bands (10 ± 1 bands) of 2 mm were manually excised. After reduction, alkylation, and trypsin digestion, peptides were extracted using 60% acetonitrile and 0.1% HCOOH for 1 hour at room temperature.

For biopsies infected via syringe inoculation, we suspended peptides in 200 µL of 0.1% HCOOH after evaporation, and nanoLC–MS/MS analyses (1 µL injected) were performed on a nano-ACQUITY UPLC (UltraPerformance Liquid Chromatography) system (Waters, Milford, MA, USA) hyphenated to a quadrupole time-of-flight MaXis4G (Bruker Daltonics, Bremen, Germany) as previously described [25] with the following modifications: elution gradient of 6–35% solvent B over 28 min at 60 °C and external mass calibration in the mass range of 100–2200 *m/z* (scan speed 25 Hz). For tandem MS experiments, acquisition was performed by sequentially selecting the maximum of precursors for a cycle time of 3.5 s, with a preference for multiply charged ions and strict exclusion of monocharged ions. Acquisition speed in MS/MS was adjusted according to the precursor intensity. Selected ions were excluded for 1 min and optionally reconsidered if the measured intensity was three times higher than the previously measured intensity. Mass data analysis was performed as previously described [22,26], except for a 25 ppm parent mass tolerance, a 0.05 Da fragment mass tolerance, and a maximum of one missed cleavage.

For biopsies infected via tick bites, we suspended peptides in 50 µL of 0.1% HCOOH after evaporation, and nanoLC–MS/MS analyses (3 µL injected) were performed on a nano-ACQUITY UPLC system (Waters, Milford, MA, USA) hyphenated to a Q-Exactive Plus mass spectrometer (Thermo Scientific, Bremen, Germany) as previously described [26], except for an elution gradient of 5–35% solvent B over 60 min, then 35–90% solvent B over 1 min, and capillary voltage set to 1.6 kV at 250 °C. Mass data analysis was performed as previously described [26], and searches were performed against an inhouse-generated database containing all protein sequences of *B. afzelii* Pko (10 October 2016, 2186 entries) and mouse (10 October 2016, 16,806 entries) (extracted from NCBInr and UniProtKB/Swiss-Prot, respectively).

For all biopsies, a target-decoy database search allowed us to control the false-positive identification rate, which was set at 1% with a minimum of one peptide per protein. All results were loaded into Scaffold software (Proteome Software, Portland, OR, USA) to validate peptide identifications.

### 2.4. In Silico Evaluation of Protein Polymorphism Among Borrelia burgdorferi Coding DNA Sequences

We performed the analysis of protein variability in the coding DNA sequences (CDs) of proteins selected at the early cutaneous phase described in *B. burgdorferi* either using translated sequences of the validated subset of UniProtKB-Swiss-Prot or annotated genes in UniprotKB-TrEMBL database, among the 15 publicly available *B. burgdorferi* genomes curated on the website borreliabase.org [27]. These *Borrelia* genomes have a taxonomic identification at the species level confirmed by phylogenomics. Sequence comparisons with reference protein sequences were performed using bidirectional protein-protein BLAST (BlastP) sequence comparison of translated open reading frames. Proteins with amino acid sequence similarities ≥65% and E-value ≤10^−10^ were considered homologous [28].

### 2.5. Ethics

The protocols carried out in this study were approved by and complied with the requirements of the CREMEAS Committee on the Ethics of Animal Experiments of the University of Strasbourg (Comité Régional d’Ethique en Matière d’Expérimentation Animale Strasbourg—Ministry Permit Number: APAFIS 2015062414395551) in the animal facilities D-67-482-34.

### 2.6. Statistical Analyses

Statistical analyses between the various groups were made with Fisher’s exact test to compare proportions, a non-parametric Kruskal–Wallis test, or a Mann–Whitney test when there were only two groups to compare. A *p*-value of 0.05 was considered statistically significant. These statistical analyses were performed with Prism 7 software v7.0a (Graphpad, La Jolla, CA, USA). Exact confidence interval of a frequency, i.e., binomial confidence interval, was determined for each percentage according to the Clopper–Pearson method using the web server http://statpages.info/confint.html.

## 3. Results

### 3.1. Efficacy of Infection and Kinetics of Bacterial Multiplication in the Mouse Skin for the Three B. burgdorferi ss Strains

We first evaluated the efficacy of *Borrelia* transmission in mice by detection of *B. burgdorferi* DNA (*flaB* gene) in the skin at the inoculation site after 3, 5, 7, or 15 days post-infection (Table 1). No significant difference was observed in the rate of positivity between the three studied strains at each time point (Fisher’s exact test, *p*-value ≥ 0.1). Maximum positive detections in the skin samples of mice were observed on day 7 post-inoculation for the three *B. burgdorferi* strains, reaching 100% of tested animals. These results also indicate that infection was effective with a syringe inoculation of 10^3^ *B. burgdorferi* spirochetes in the skin of mice.

**Figure 1:**
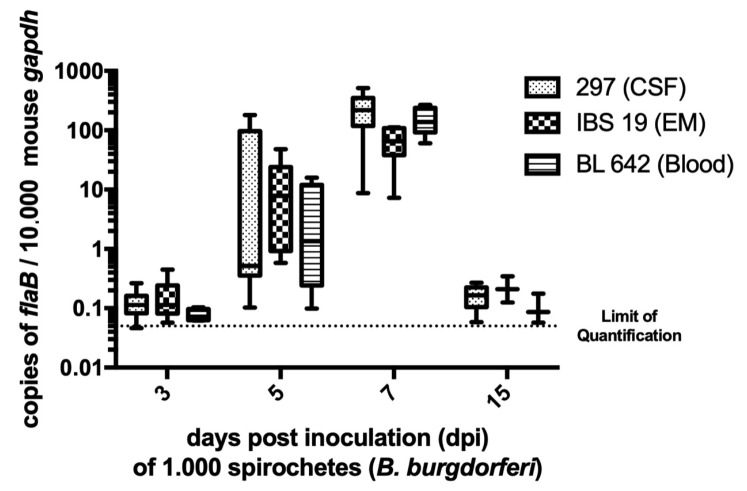
*Borrelia* load in the mouse skin. The spirochetal burden in the skin at the inoculation site was measured by quantitative PCR for the *Borrelia* flagellin (*flaB*) gene and normalized to copies of mouse glyceraldehyde-3-phosphate dehydrogenase (*gapdh*). Values presented in box plot correspond to relative quantification in each positive mouse skin (all the mice referred to as positive in Table 1). In each box plot, the box embraces values between the first and third quartile, the median is a line, the higher error bar corresponds to the maximum value, and the lower error bar corresponds to the minimum value. Limit of quantification was around 0.05 *flagellin* copies per 10.000 murine *gapdh*. Medians of bacterial load were statistically compared [1] within strains at the same day and [2] for a same strain at each time post-inoculation. The bacterial load reached maximum density for the three strains on day 7 (*p*-values < 0.05). Values of bacterial load were not statistically different between strains at each time point, except for strain IBS 19 that exhibited a lower median value of spirochetal load on day 7 (*p*-value < 0.01). Abbreviations: CSF, cerebrospinal fluid; EM, erythema migrans.

We then quantified *Borrelia* at the inoculation site at the various time points of the kinetics study by real-time PCR targeting *flagellin* gene with a standard curve. The number of *flaB* copies in samples was standardized to 10,000 copies of the housekeeping murine gene *gapdh* (Figure 1). Three- and 15-days post-inoculation (dpi), spirochetes were near the limit of quantification for all three *B. burgdorferi* strains (0.05 *flagellin* copies per 10,000 murine *gapdh*). On day 5, for some skin samples, bacterial load was detected but it did not exceed 200 *flagellin* copies per 10,000 murine *gapdh*. The bacterial load reached maximum density for the three strains on day 7 (*p*-values < 0.05) with median values of 219 (min. 9; max. 515), 65 (min. 7; max. 111), and 138 (min. 60; max. 268) *flagellin* copies per 10,000 murine *gapdh* for strains 297, IBS 19, and BL 642, respectively. Altogether, the kinetics of bacterial load suggested that the spirochetal multiplication peak in mouse skin occurs approximately 7 days after inoculation under such experimental conditions. Values of bacterial loads were not statistically different between strains at each time point, except for strain IBS 19, which exhibited a lower median value of spirochetal load on day 7 (*p*-value < 0.01), but its kinetic profile and the scale of values were comparable to strains 297 and BL 642 (see the median values indicated above).

### 3.2. Detection of Borrelia Proteins on Day 7 by Proteomics

Using non-targeted mass spectrometry on mice infected via syringe inoculation, we selected samples of infected skin on day 7—i.e., the peak of *Borrelia* multiplication in the skin—to increase the probability of finding a significant amount of bacterial proteins. We identified between two and nine proteins of *Borrelia burgdorferi* ss among an average of 1620 mouse proteins with flagellin, OspC, and GAPDH, the most frequently detected proteins (Table 2). According to *Borrelia* strains, we detected additional proteins such as chaperonin (GroEL), enolase, lipoprotein BbA36, and p66. 

Interestingly, when mice were infected by ticks (either with the ones infected under laboratory conditions or collected in the field), we also detected OspC and flagellin of *B. afzelii*. This is the predominant species circulating in ticks and isolated in patients in Europe.

Thanks to the Q-Orbitrap mass spectrometer, we identified additional proteins (average of 5920 mouse proteins) when mice were infected through an infectious tick bite, and we also detected 12 additional proteins of *Borrelia* (Table 2). Given the difference in the two types of equipment, the detection of additional bacterial proteins cannot be strictly assigned to the inoculation route, but also (if not exclusively) to the enhanced sensitivity of Q-Orbitrap mass spectrometer.

### 3.3. Peptidic Polymorphism of the Selected Coding DNA Sequences Among B. burgdorferi ss Genomes

We evaluated peptide variability of the coding DNA sequences (CDSs) for the six *Borrelia* proteins identified by proteomics (major flagellar protein FlaB, glyceraldehyde-3-phosphate dehydrogenase (GAPDH), 60 kDa chaperonin (GroEL), enolase, lipoprotein BbA36, and outer surface protein C (OspC). To this end, we performed an *in silico* comparison between the coding DNA peptide sequences from 15 *B. burgdorferi* ss genomes on the webserver borreliabase.org. The location of genes encoding for flagellin, GAPDH, GroEL, and enolase proteins is chromosomal, whereas lipoprotein BbA36 and OspC are plasmid-encoded in the lp54 and cp26 plasmids, respectively. The most conserved peptide sequences corresponded to the flagellin, GAPDH, GroEL, and enolase proteins, with homologs detected in all 15 genomes and excellent features of identity (≥99%) and similarity (100%) between strains, with the exception of the enolase CDS in strain 29805 that appeared truncated (Table 3). Homologs of lipoprotein BbA36 were detected in 13 *B. burgdorferi* ss strains and not in two strains for which the lp54 plasmid had not been sequenced. Identity and similarity features were high (≥97%), and we observed minor differences from the reference sequence including a supplementary amino acid detected in six strains and differences in the first eight amino acids that were specific to strain B31. OspC peptide sequences were significantly more polymorphic with 82 to 88% identity and 74 to 81% similarity for the homologous sequences detected in the 12 genomes harboring cp26 and other than the reference genome (Table 3).

## 4. Discussion

Although various antigens have been identified as vaccine candidates for Lyme borreliosis, none have been particularly effective in protecting animals and humans [29]. It is well-established that the antibody response can control the bacterial infection, but it is not long-lasting. The complexity of this immune response against *Borrelia* is linked to high surface antigen variation, especially with up- and downregulation of VlsE (Variable major protein-like sequence, Expressed) [30], and inhibition of complement lysis pathway [31]. Interestingly, recent data show that *Borrelia* infection actually induces a temporary immunosuppression, which explains the lack of long-term immunity for this disease [32]. Presently, several vaccines have been developed and marketed in veterinary medicine for dogs. They are based on whole-lysate antigens or recombinant OspC or OspA protein. In humans, clinical trials are underway in Europe using a modified OspA antigen [33]. The vaccine is based on six different OspA serotypes of different *Borrelia* species occurring in the United States and Europe and formulated with aluminum hydroxide [34]. This vaccine (VLA15), previously tested in a mouse model, induced a good protection after three immunizations against a challenge of *Borrelia* inoculated using a syringe or via infected tick bites [35].

Up to now, this unsuccessful strategy to get an effective vaccine against Lyme borreliosis could be either due to inefficient vaccine candidates, or failure in the delivery system of the vaccine, or both, in terms of the ability to elicit a protective immune response. As the skin is an essential interface in VBDs, we found the development of a new strategy to identify relevant *Borrelia* proteins expressed in the skin during the early process of transmission to be particularly interesting. Indeed, antibodies directed against these proteins would neutralize bacteria before they further disseminate in deep organs: the heart, the joints, or the central nervous system. We decided to use the discovery proteomics approach, Ge-LC–MS/MS, to identify *Borrelia* proteins in the skin of infected mice after syringe inoculation or after an infectious tick bite. The C3H mouse model of Lyme borreliosis has been shown to be very effective in analyzing mechanisms of *Borrelia* transmission and persistence [26,36]. In this study, we showed that non-targeted mass spectrometry allows for the identification of at least 30 *Borrelia* proteins. Some of them have already been described as potential vaccine candidates, such as OspC, enolase, and DbpA.

As *Borreliae* are highly diverse [37], some bacterial proteins were only detected in some *Borrelia* strains, as shown, for example, for *Borrelia* glycosaminoglycan binding protein (Bgp), which is present only in the strain BL642, or GAPDH present in strains 297c4 and BL642, or GroEl protein detected in *B. burgdorferi* ss strain 297 and in *B. afzelii* from mice infected through the bite of field-collected ticks.

The proteins OspC and FlaB (p41) are immunodominant antigens of *Borrelia* that are implicated in the initial human immune response against spirochetes [38]. Mainly composed of the non-glycosylated major flagellar protein FlaB, the periplasmic flagellar filaments are essential in the motility and mammal infectivity of *B. burgdorferi* [39]. Proteomic assays highlighted that FlaB was abundant in the skin at the inoculation site, with a higher load 7 days after inoculation, i.e., at the time of the putative multiplication of spirochetes, as confirmed by quantification of the *FlaB* transcript. This relative abundance during the early cutaneous phase of the infection and the remarkable peptide sequence homogeneity between strains that we reported (no polymorphism from 15 *B. burgdorferi* ss genomes) may suggest that FlaB represents a relevant vaccine candidate as purified recombinant protein. However, Fikrig et al. [40] showed that FlaB had no role in the protective immunity of C3H mice against spirochetal infection by *B. burgdorferi*, unlike OspA and OspB. However, bacterial flagellin has recently been studied as a potential adjuvant in the development of novel vaccines [41]. As a vaccine candidate, flagellin has been tested as a protein target (e.g., in the *Campylobacter jejuni* vaccination of poultry [42]) or as an adjuvant (e.g., in the *Clostridium difficile* vaccination [43]). In this context, a thorough study of FlaB immunogenicity in Lyme borreliosis deserves to be performed using the protein as adjuvant and/or in combination with other *Borrelia* antigens.

Outer surface protein OspC is a well-known lipoprotein of *B. burgdorferi* sensu lato (sl) induced during nymphal tick feeding. It is essential for the tick-to-mammal transmission of the bacteria [44]. It is therefore not surprising to detect it in the skin by proteomics. However, the high rate of peptide polymorphism observed between strains obtained in our study confirmed the hypervariability of this protein reported by previous works [29,45]. Due to this high strain specificity, OspC cannot be a good vaccine candidate.

Enolase is an enzyme involved in glycolysis. It constitutes a plasminogen receptor and mediates the activation of plasmin and extracellular matrix degradation [46]. As a protein expressed on cancer cells to facilitate their dissemination, this protein is very well studied and has been proposed as a potential vaccine candidate in cancer [47]. It is also a ubiquitous protein in animals. *Borrelia* is known to bind to plasminogen via this protein to facilitate its dissemination through host tissues. Experiments of mouse immunization with recombinant enolase did not induce protective immunity against subsequent *B. burgdorferi* infection. However, mice immunized with this protein reduced pathogen survival within feeding ticks [48]. In our study, the enolase peptides were weakly detected in the various *Borrelia* strains and species.

GroEL is a 60-kDa heat shock protein that prevents misfolding and promotes the refolding and proper assembly of unfolded polypeptides generated under stress conditions. It mainly represents a cytosolic protein with a few proteins inserted in inner membrane [49]. As a ubiquitous protein in both prokaryotes and eukaryotes and as a highly conserved protein, it has been used to genotype various *B. burgdorferi* sensu lato strains [50]. Interestingly, it has been detected in the skin of mice infected with *B. burgdorferi* ss and *B. afzelii*, irrespective of the inoculation mode.

GAPDH (glyceraldehyde-3-phosphate dehydrogenase) has an enzymatic role in the glycolytic pathway. It has been detected by 2D gel (2-DE) immunoblotting with matrix-assisted laser desorption/ionization mass spectrometry (MALDI-MS). Antigens of *B. garinii* were analyzed for their reactivity with sera from patients at the early and late stage of Lyme borreliosis. Among the 20 antigens identified, GAPDH was detected [51]. In our study, this protein was only detected after syringe inoculation of *Borrelia*.

Elongation factor Tu is a cytosolic protein, just like enolase, and can be localized on the surface of bacteria. It has been shown to be very immunogenic and to induce antibodies in patients. However, this protein has not proved protective after immunization in mice [52]. Interestingly, in our study, we found this protein in the mouse skin after syringe inoculation of *Borrelia burgdorferi* ss, but also in mice naturally infected with *B. afzelii*-infected ticks.

Vaccines are the most effective way of preventing VBDs [53]. Presently, only a few vaccines are available. They target viral pathogens such as the vaccines against tick-borne encephalitis, yellow fever, and Japanese encephalitis. It points out to weaknesses in the design of such vaccines, especially against bacteria and parasites. Pitfalls have to be identified and could be due to an incorrect identification/selection of vaccine candidates, an inefficient adjuvant, and/or a poor delivery system of vaccination. *Borrelia* antigens detected in the skin by mass spectrometry might constitute a reliable approach to identify vaccine candidates and to elaborate cocktails of antigens that are immunogenic and protective. We selected the skin samples of infected mice on day 7 as we know that *Borrelia* multiplies very actively on day 7 [23]. In the design of vaccine for vector-borne diseases, the adjuvant and the delivery system also play a major role to elicit strong immunity. Indeed, the skin is a complex immune organ and it constitutes a persistent site for several vector-borne pathogens [54,55]. The skin is also a key interface of specific immune response during inoculation of these pathogens [21]. It constitutes the best interface to address the specific interactions of the host immune system and the pathogen proteome in VBDs. Besides the technique of mass spectrometry to identify vaccine candidates in the skin, data bank searches are also essential to overcome the problems of gene variability for these vaccine candidates.

New concepts have emerged in the development of vaccines with the advances of “omics” and reverse vaccinology [56,57]. In the field of proteomics, various techniques have been suggested to identify potential vaccine candidates. Some studied the surface proteome of *Borrelia* [58]. However, the three studied *Borrelia* species were issued from in vitro culture. Interactome studies have also been performed [59], but they once again relied on in vitro study interaction. It is now well-documented that *Borrelia* adapts to its various hosts during its enzootic cycle through modifications of its transcriptome [60,61]. It is therefore essential to work in vivo with animal models, mimicking as closely as possible the natural environment of pathogens. The skin thus constitutes an excellent organ to identify *Borrelia* proteins essential for the early transmission of this TBD. Thanks to its specific immunity, temperature, and microbiome, the skin also represents a peculiar environment [62] where some pathogens can persist for months by inducing immune tolerance [26].

## 5. Conclusions

The identification of good vaccine candidates is key to the development of a vaccine, and so is the choice of the adjuvants and antigen delivery system in order to induce protective immunity. Besides aluminum hydroxide, which is largely used in vaccines, new adjuvants are being developed to improve the strength and quality of the immune response such as AS01 adjuvant [63]. Innovative techniques have also been developed lately to better deliver antigens in the outermost layer of the skin by microneedles [64]. This has already been tested in human clinical trials for immunization against influenza virus, with promising results [65].

Facing all the unsuccessful strategies developed in the field of vaccines against VBDs, it is essential to reevaluate the various techniques used to identify vaccine candidates. It is also essential to better understand skin immunity against vector-borne pathogens that are inoculated, which multiply intensively and persist in the skin. In this context, a broader multidisciplinary approach in vaccinology instead of the approach used up until now should open up new avenues to control VBDs.

## Figures and Tables

**Table 1:** Detection of *Borrelia burgdorferi* sensu stricto (ss) DNA in the skin of infected mice 3, 5, 7, or 15 days post-inoculation (dpi). This table presents the results of all the mice included in the study. Abbreviations: CI 95%, 95% confidence interval; PCR, polymerase chain reaction.

Days Post-Inoculation (dpi)	Strains	Positive PCR Amplification	
		Positive Reactions/No. of Tested Animals	Rate of Positivity (CI 95%)
3 dpi	297	14/19	74% (49–91)
	IBS 19	6/11	55% (23–83)
	BL 642	4/11	36% (11–69)
5 dpi	297	16/17	94% (71–100)
	IBS 19	8/8	100% (63–100)
	BL 642	6/7	86% (42–100)
7 dpi	297	15/15	100% (78–100)
	IBS 19	10/10	100% (69–100)
	BL 642	6/6	100% (54–100)
15 dpi	297	6/13	46% (19–75)
	IBS 19	3/7	43% (10–82)
	BL 642	3/7	43% (10–82)

**Table 2:** Identification of bacterial proteins in mouse skin infected by intradermal inoculation of different *Borrelia burgdorferi* strains, or via infectious tick bite. Proteins were identified using Mascot algorithm. Numbers of identified peptides are given.

Protein Name	Accession Number (*Borrelia burgdorferi* B31)	Accession Number (*Borrelia burgdorferi* Pko)	Infection via Syringe Inoculation *Borrelia burgdorferi* Strain *(Spirochete Density Flagellin/10^4^gapdh)*									Infection via Tick Bite *(Spirochete Density Flagellin/10^4^gapdh)*	
			297c4 *(192)*	297c4 *(106)*	297n *(346)*	IBS19 *(85)*	IBS19 *(136)*	IBS19 *(166)*	BL642 *(157)*	BL642 *(227)*	BL642 *(268)*	*B. Afzelii* NE4049 *(66)*	Field-Collected Ticks *(534)*
Flagellin	gi|120230	gi|111114970	7	8	7		2	2	3	3	2	4	8
Osp C	gi|3914248	gi|111074137	3	2	3	1	2	2	2	1	1	4	1
GAPDH	gi|238828321 gi|3915702		1	1	1					1	1		
Glycosaminoglycan binding Protein (Bgp)	gi|15594933								1	1	1		
GroEL	gi|229553917	gi|123145654	1	1									2
p66	gi|15594948		1										
Fructose-bisphosphate aldolase	gi|6686370	gi|111115272											1
DbpA	gi|17373807		1										
DbpB	gi|327507700				2								
Elongation factor 4	gi|15594434 gi|6016495					1							
Elongation factor Tu	gi|1706598	gi|123341337	1							1		1	2
Enolase	gi|3913583		1										
Lipoprotein BbA36	gi|365823350				2								
L-lactate dehydrogenase	gi|15594433 gi|17367476				1								
hypothetical protein BB_0363	gi|15594708									1			
hypothetical protein BB_0563	gi|365992369		1	1									
hypothetical protein BB_F14	gi|365823340			1	1								
hypothetical protein BB_J48	gi|364556751										1		
hypothetical protein BAPKO_4515		gi|117621815											1
hypothetical protein BAPKO_0028		gi|111114851											1
hypothetical protein BAPKO_0593		gi|111115391											1
hypothetical protein BAPKO_2500		gi|117621647										1	
50S ribosomal protein L7/L12		gi|123046997											1
30S ribosomal protein S16		gi|123145645										1	1
Neutrophil activating protein		gi|111115523											2
Phosphoglyceromutase		gi|123145651											1
Flagellar filament outer layer protein		gi|111115501											1
ATP-dependent protease		gi|111115078										1	1
Periplasmic oligopeptide-binding protein		gi|111115153											1
Chemotaxis protein methyltransferase		gi|111114863										1	
Number of *Borrelia* proteins			9	6	7	2	2	2	3	6	5	7	15
Number of mouse proteins			1831	1791	1824	1486	1617	1550	1702	1351	1426	5916	5926

**Table 3:** Peptide variability of six protein-coding sequences in the species *B. burgdorferi* sensu stricto. The 15 *B. burgdorferi* sensu stricto strains studied were B31, CA382, 64b, ZS7, BOL26, WI91-23, 29805, N40, JD1, 156a, 94a, CA-11.2A, 72a, 118a, and CA8. Abbreviations: aa, amino acids; CDS, coding DNA sequence; Id., identity; S., similarity; WGS, whole genome sequences.

Protein (Length)	Protein Accession Number (B31)	Gene Accession Number (B31)	CDS Location	Peptidic Variability Among the CDS of 15 *B. burgdorferi* Sensu Stricto Strains
FlaB (336 aa)	SwissProt P11089	Genbank BB_0147	chromosome	100% S./100% Id.: 15/15 strains
GAPDH (335 aa)	SwissProt P46795	Genbank BB_0057	chromosome	100% S./100% Id.: 14/15 strains 100% S./99% Id.: 1/15 strain
GroEL (545 aa)	SwissProt P0C923	Genbank BB_0649	chromosome	100% S./100% Id.: 15/15 strains 1 supplementary aa in 1 strain
Enolase (433 aa)	SwissProt O51312	Genbank BB_0337	chromosome	100% S./100% Id.: 12/15 strains 100% S./99% Id.: 2/15 strains Truncated peptidic CDS in the 29805 strain (from 219th to 273th aa)
Lipoprotein BbA36 (212 aa)	TrEmbl O50929	Genbank BB_A36	lp54 plasmid(13/15 strains)	100% S./100% Id.: 2/13 strains 100% S./99% Id.: 2/13 strains 97–99% S./97–99% Id.: 9/13 strains Not found in 2 WGS without sequenced lp54 plasmid 8 first aa not annotated in the 12 strains other than B31 1 supplementary Asparagin between the 80th and 81th residue of the reference sequence in 6/13 strains
Outer surface protein C (210 aa)	SwissProt Q07337	Genbank BB_B19	cp26 plasmid(13/15 strains)	100% S./100% Id.: 1/13 strains 82–88% S./74–81% Id.: 12/13 strains Not found in 2 WGS without sequenced cp26 plasmid
